# Seroexposure to Zoonotic *Anaplasma* and *Borrelia* in Dogs and Horses That Are in Contact with Vulnerable People in Italy

**DOI:** 10.3390/pathogens12030470

**Published:** 2023-03-16

**Authors:** Donato Traversa, Piermarino Milillo, Raffaella Maggi, Giulia Simonato, Angela Di Cesare, Carlo Pezzuto, Marika Grillini, Simone Morelli, Mariasole Colombo, Alessandra Passarelli, Antonio Grassano, Paola Serio, Michele Losurdo, Roberto Brueckmann

**Affiliations:** 1 Department of Veterinary Medicine, University of Teramo, 64100 Teramo, Italy; 2 Azienda Sanitaria Locale (A.S.L.), 75100 Matera, Italy; 3 Freelance Veterinary Practitioner, 00189 Rome, Italy; 4 Department of Animal Medicine, Production and Health, University of Padua, 35020 Legnaro, Italy; 5 Ambulatorio Veterinario Pezzuto Carlo/Piano Noemi, 86010 Campobasso, Italy; 6 Clinica Veterinaria Città di Bari, 70125 Bari, Italy; 7 Freelance Veterinary Practitioner, 75020 Nova Siri, Italy; 8 EUROIMMUN Medizinische Labordiagnostika AG, 23560 Lübeck, Germany

**Keywords:** Anaplasmosis, borreliosis, zoonosis, vulnerable people

## Abstract

Equine and canine anaplasmosis and borreliosis are major tick-borne zoonotic diseases caused by *Anaplasma phagocytophilum* and various species of *Borrelia* (the most important being *Borrelia burgdorferi* s.l.), respectively. This study evaluated the seroexposure to *Anaplasma* and *Borrelia* in dogs and horses used in Animal-Assisted Interventions or living in contact with children, elderly people or immunocompromised persons. A total of 150 horses and 150 dogs living in Italy were equally divided into clinically healthy animals and animals with at least one clinical sign compatible with borreliosis and/or anaplasmosis (present at clinical examination or reported in the medical history). Serum samples were tested with ELISA and immunoblot for the presence of antibodies against *A. phagocytophilum* and *B. burgdorferi* s.l., and the association between seropositivity and possible risk factors was analyzed using multivariate and univariate tests. Overall, 13 dogs (8.7%) and 19 horses (12.7%) were positive for at least one of the two pathogens. In addition, 1 dog (0.7%) and 12 horses (8%) were positive for antibodies against *A. phagocytophilum*, while 12 dogs (8.0%) and 10 horses (6.7%) had antibodies against *B. burgdorferi* s.l. Tick infestation in the medical history of the dogs was significantly associated with seropositivity to at least one pathogen (*p* = 0.027; OR 7.398). These results indicate that, in Italy, ticks infected with *A. phagocytophilum* and/or *B. burgdorferi* circulate in places where horses and dogs are in contact with people at risk of developing severe diseases. Awareness should be increased, and adequate control plans need to be developed to protect human and animal health, especially where vulnerable, at-risk individuals are concerned.

## 1. Introduction

Tick-borne diseases (TBDs) have a great impact on the health and welfare of animals and humans. Among the different bacteria transmitted by ticks, *Anaplasma phagocytophilum* and *Borrelia burgdorferi* sensu latu (s.l.) cause different illnesses in animals and have significant zoonotic potential, with possibly severe and fatal outcomes [1,2,3,4,5,6]. In particular, anaplasmosis and borreliosis have clinical relevance in dogs and horses, which contribute to the epidemiological maintenance of these diseases.

Most dogs infected with *A. phagocytophilum* or *B. burgdorferi* s.l. remain clinically healthy, although they are subclinically infected and thus serve as a source of infection for ticks [7,8,9,10]. In addition, when clinical signs appear in infected animals, they are often non-specific, making diagnosis even more difficult [11,12]. Clinical signs that can be present include lethargy, fever, anorexia, musculoskeletal disorders, neurological signs, uveitis, cough, polydipsia, gastrointestinal signs and bleeding disorders in canine anaplasmosis [7,13,14]. Different *A. phagocytophilum* strains have been described, with different pathogenic potential [14] Canine borreliosis can manifest with lameness, arthritis, fever, glomerulonephritis proteinuria, hyperazotemia, peripheral edema, and body cavity effusions [13].

Horses infected with *A. phagocytophilum* may display fever, lack of appetite, weakness, stiff gait, lameness, weight loss, limb swelling, gastrointestinal and neurological signs [15]. Data on clinical equine borreliosis are limited, though neurological signs, uveitis, joint effusion and cardiac arrythmias have been reported [9].

In humans, *A. phagocytophilum* causes a febrile illness (“granulocytic anaplasmosis”) characterized by headache, myalgia, and gastrointestinal disorders. Other clinical signs, e.g., conjunctivitis, arthralgia, lymphadenomegaly may be present [16,17,18]. The pathogenic potential may differ among strains of *A. phagocytophilum* and human anaplasmosis is more frequent in US than Europe [14].

Human Lyme disease, caused by *B. burgdorferi* s.l., is a well-known disease distributed throughout the northern hemisphere and characterized by severe neurological signs, skin lesions (e.g., erythema migrans), arthritis, and heart diseases [19].

Dogs and horses are among the most popular pets and animals used in Animal-Assisted Interventions (AAI) and are in close contact with children or other vulnerable patients [20]. Despite the abundance of epidemiological data on canine and equine TBDs [14,21,22], specific information on the exposure to zoonotic anaplasmosis and borreliosis from dogs and horses living in contact with vulnerable people is scarce. In fact, the overlap of clinical signs in animals suffering from anaplasmosis or borreliosis with other diseases and the limitations of available diagnostic options often lead to an underestimation of these TBDs [7,14,23]. From an epidemiological point of view, knowledge of animal exposure provides evidence of the presence of infected ticks in a given setting and potential risks to animals and humans.

To date, there is no reported case in which a tick-borne disease such as Lyme disease or anaplasmosis has been transmitted from an infected pet to humans. However, the risk of indirect zoonotic disease transmission by pets in AAI via infected ticks should be considered [24]. In addition, these infections can have serious consequences for pets, and humans can also be indirectly affected. Every infection represents a stressor for the organism, and even subclinical infections are fought by the immune system and require various physiological reactions. A central role in controlling these processes is played by the Hypothalamic–Pituitary–Adrenal (HPA) axis, which is largely responsible for the physiological stress response of the organism and modulates immune responses [25,26]. Although infections promote an increased release of stress hormones through cytokines [27,28], not all of them (e.g., Lyme disease) manifest in measurable physiological responses such as fever or lameness. Often, infections cause behavioral changes in affected animals, e.g., increased petulance or lethargy [29,30]. These are not always recognized as signs of a disease by animal owners, which is important in the case of animals involved in the AAI, which may show unexpected stress reactions, e.g., aggressive behavior [31,32,33,34].

The current scarcity of data on this topic calls for studies aiming at evaluating the risk of exposure to zoonotic anaplasmosis and borreliosis in dogs and horses that live or are in contact with persons at risk. Therefore, the present study investigated the seropositivity against *A. phagocytophilum* and *B. burgdorferi* s.l. in dogs and horses with or without compatible clinical signs that are living or in contact with people susceptible to developing severe diseases.

## 2. Materials and Methods

### 2.1. Study Animals

A total of 150 dogs and 150 horses involved in AAI and/or living in daily contact with children, elderly people or immunocompromised persons was selected in regions in northern, central, and southern Italy. All animals were taken by their owners to their veterinarians for routine examinations or because they showed clinical signs suggestive of TBDs. A serum sample was collected from each animal during routine preventive programs or for procedures required by the attending veterinarians. Written informed consent was obtained from all owners.

Depending on the presence/absence of compatible clinical signs, the animals were equally divided (75 animals for each group) as follows: clinically healthy dogs and horses, and dogs and horses presenting at least one clinical sign/abnormal laboratory finding (detected during clinical examination or reported by the owner) compatible with the disease caused by *A. phagocytophilum* and/or *B. burgdorferi* s.l. (Table 1 and Table 2). Healthy and sick animals were homogeneously distributed among study sites, i.e., 25 healthy and 25 sick dogs and horses, respectively, for each study site.

For each animal, information on age, sex, lifestyle, habitat, anamnesis, presence/absence of clinical signs or alterations was recorded. In addition, the history of antiparasitic treatment and the presence of ticks found on the animal during the clinical examination or in the history were recorded.

### 2.2. Serological Analysis

All samples were tested for antibodies against *A. phagocytophilum* and *B. burgdorferi* s.l. using enzyme-linked immunosorbent assays (ELISA). Positive and borderline ELISA results were confirmed by immunoblot. Details of the immunoassays from EUROIMMUN Medizinische Labordiagnostika AG, Luebeck, Germany used are listed in Table 3.

The ELISA kits used here provide semiquantitative in vitro determination of canine or equine antibodies of the immunoglobulin class IgG or IgM against the respective antigen in serum or plasma. Results are expressed as the ratio between the extinction of the sample/control and the extinction of the calibrator. EUROLINE kits used for immunoblotting provide qualitative in vitro determination of antibodies of the immunoglobulin class IgG or IgM in serum or plasma.

Incubation, evaluation, and interpretation were performed according to the respective instructions for use of the manufacturer. Evaluation of the EUROLINE blots was performed with the EUROLineScan software (EUROIMMUN, Lubeck, Germany). In general, ELISA assays were used as screening tests, whereas positive or borderline results were analyzed by immunoblot as confirmatory tests. In dogs, three ELISAs and three immunoblots were used, differing in individual specifications. The Anti-*Borrelia* ELISA IgG and IgM use full antigens of *Borrelia* to detect either IgM (early infection phase) or IgG (late infection phase) antibodies. In contrast, the Anti-VlsE ELISA Dog uses only the recombinant VlsE antigen to detect IgG antibodies against *Borrelia*. Due to its highly specific antigen, the VlsE ELISA could be used without a confirmatory test of positive/borderline results. However, in order to cover the whole antibody spectrum and not to miss any seropositive animal, all tests were performed in parallel. In terms of the immunoblots used, the Anti-*Borrelia* EUROLINE IgM and IgG were used as confirmatory tests using specific and recombinant antigens from *Borrelia* (see Table 3). The third test (EUROLINE Tick-Borne Profile dog) uses common and highly specific and recombinant *Borrelia*-antigens but also recombinant antigens of *Anaplasma* and TBEV. Therefore, this immunoblot allows differentiation between three of the most common tick-borne diseases.

For horses, only the IgG Anti-*Borrelia* ELISA and Anti-*Borrelia* EUROLINE as well as the EUROLINE Tick-Borne Profile 1 Horse (IgG) were used, as no specific IgM assay is available.

### 2.3. Statistical Analysis

Multivariate (Binomial Logistic Regression) and univariate (Fisher’s exact test) statistical analyses were performed to evaluate statistically significant associations (*p* < 0.05) between possible risk factors (e.g., lifestyle, presence/absence of clinical signs, antiparasitic treatments, presence of ticks or history of tick infestations) and seropositivity to *A. phagocytophilum* and/or *B. burgdorferi* s.l. The strength of association between risk factors and presence of antibodies was calculated using the odds ratio (OR) and the 95% Confidence Interval (95% CI). The statistical analysis was performed using GraphPad Prism 9 (GraphPad Software, LLC, La Jolla, CA, USA).

## 3. Results

Overall, 13 dogs (8.7%) and 19 horses (12.7%) were positive for at least one pathogen (Table 4). One dog (0.7%) from northern Italy and 12 horses (8%), i.e., 3 from northern Italy, 5 from central Italy and 4 from southern Italy, tested positive for *A. phagocytophilum*. Twelve dogs (8%), i.e., 2 from northern Italy, 1 from central Italy, and 9 from southern Italy, as well as 10 horses (6.7%), i.e., 6 from northern Italy, 2 from central Italy and 2 from southern Italy, showed antibodies against *B. burgdorferi* s.l. Of these dogs, IgM class antibodies were detected in 11 animals, while IgG class antibodies were detected in only one animal. Equine samples were analyzed using only IgG assays. Three horses (2%) were seropositive for both *A. phagocytophilum* and *B. burgdorferi* s.l.

In total, 16 seropositive animals (5.3%) had clinical signs compatible with a TBD on examination or in their medical history. The only dog positive for *A. phagocytophilum* showed no clinical signs. Of the dogs in which only antibodies to *B. burgdorferi* s.l. were found, 5 out of 12 (41.7%) presented clinical signs (Table 5) on clinical examination. In horses with antibodies against *B. burgdorferi* s.l. only 2 out of 7 (28.6%) showed clinical signs (Table 6). In addition, 7 out of 9 (77.8%) horses positive only for *A. phagocytophilum* displayed clinical alterations on examination. Of them, one horse had also clinical signs reported in its medical history. Two out of three horses with antibodies against both *A. phagocytophilum* and *B. burgdorferi* s.l. showed clinical signs on clinical examination. Of the animals that were seropositive to at least one pathogen, five (i.e., two dogs and three horses) displayed clinical signs related to the locomotor system, while four (three dogs and one horse) presented fever and three (two dogs and one horse) were lethargic.

Overall, 135/150 dogs (90%) and 111/150 horses (74%) received treatments for ectoparasites. In particular, 38 dogs (25.3%) and 21 horses (14%) received a year-round protection, 66 dogs (44%) and 34 horses (22.7%) from spring to fall, 14 dogs (9.3%) and 23 horses (15.3%) only during summer and 17 dogs (11.3%) and 29 horses (19.3%) received random/irregular treatments. In 15 dogs (10%) and 39 horses (26%), the owners indicated that they did not perform any ectoparasitic treatments, and none of these animals were vaccinated against *Borrelia*.

Regarding the presence of ectoparasites, 14/150 (9.3%) and 78/150 (52%) dogs as well as 10/150 (6.6%) and 98/150 (65.3%) horses had feeding ticks on clinical examinations and in their medical history, respectively. In addition, 2 out of the 14 tick-infested dogs received ectoparasiticides throughout the year, while 1 and 3 were treated from spring to fall and in summer only, respectively; 3 received random/irregular administrations, and in 5 dogs the owners reported not using parasiticides. Meanwhile, 5 out of 10 horses with tick infestation received parasiticides only in summer, while the remaining were subjected to random/irregular administrations.

### Statistical Analysis

A statistically significant association (*p* = 0.027; OR 7.398; 95% CI = 0.22/3.78) was found between tick infestation in the medical history of dogs and seropositivity for at least one of the pathogens investigated (Table 7). No other statistically significant associations were detected in the multivariate analysis nor in Fisher’s exact test.

## 4. Discussion

This study shows that dogs and horses living or in contact with vulnerable people in regions of Italy are exposed to the bites of ticks infected with zoonotic *A. phagocytophilum* and *B. burgdorferi* s.l. To the best of the authors’ knowledge, this is the first study on the exposure to *A. phagocytophilum* and *B. burgdorferi* s.l. of dogs and horses that have close and frequent contact with children, elderly, immunocompromised or disabled subjects or that are involved in AAI. Although direct comparison with similar studies is not possible, some observations from previous surveys in terms of infection rates in other countries add new insights into these canine and equine TBDs. *Anaplasma phagocytophilum* has been found in dogs and horses in European countries with seroprevalence rates of 0.6–43.2% and of 20–22.3%, respectively [35,36,37,38]. Studies carried out in dogs in Italy showed seroprevalence rates for *A. phagocytophilum* between 3.3 and 32.8%, with higher values in the southern and central regions [39,40,41,42]. Equine anaplasmosis has been poorly investigated in Italy, where rates of ~17% were recorded more than 10 years ago [43,44]. Antibodies against *B. burgdorferi* s.l. in dogs and horses from Europe may occur with rates of up to 40–50% [21]. In Italy, seropositivity for *B. burgdorferi* s.l. in dogs has been detected with variable seroprevalence rates in northern (1.7%) [45], central (up to 1.47%) [41,45], and southern (up to 5.4%) Italy [45,46,47,48]. In horses, seropositivity for *B. burgdorferi* s.l. ranges from 7% to 24.3% in central regions [49,50]. In contrast, for northern and southern Italy, there is practically no information.

The general seroprevalence detected in this study is lower compared to previous ones, which is probably due to the categories of animals sampled here. Animals involved in AAI and/or living or in contact with children, elderly people or immunocompromised persons are supposed to be subjected to health checks and prophylactic antiparasitic treatments more frequently than the average canine and equine population. However, the seroprevalence for *B. burgdorferi* s.l. in the dogs of this study was almost identical to the seroprevalences carried out in previous studies from northern, central, and southern Italy. This is surprising, as a lower seroprevalence was also expected for *B. burgdorferi* s.l. One reason might be the use of different methodologies for the detection of antibodies against *B. burgdorferi* s.l. For example, the IDEXX SNAP 4dx test uses the C6 antigen, which is part of the highly specific VlsE antigen expressed by *B. burgdorferi* s.l. during host infection [51]. Given that IgG antibodies against the VlsE antigen are detectable in the blood of the host at an early infection stage, the detection of the corresponding antibodies is often considered as an indicator of a present infection [52]. Although the C6 antigen represents a reliable tool for investigating the infection status of dogs, the exclusive use of this antigen in the SNAP 4dx test may miss other antibodies, such as those for OspC, that are present at other infection stages [53]. In addition, antibody titers decrease over time, and C6 antibodies may no longer be detectable in the host, whereas other antibodies to *B. burgdorferi* s.l. continue to be detectable. The *Borrelia* ELISA tests used in the present study cover the broad range of antibodies against *B. burgdorferi* s.l. As already described in the results, of the 12 positive tested dogs, 11 showed IgM antibodies, whereas only 1 animal showed positive IgG antibodies (against VlsE). This may explain why the seroprevalence of *B. burgdorferi* s.l. antibodies was almost the same in the AAI dogs of this study compared with the dogs examined in other studies. In addition, the use of an immunoblot as a confirmation test facilitated the differentiation between the titers of specific antibodies against *B. burgdorferi* s.l. and thus allowed a broader statement about the “infection status” in dogs and horses. Therefore, if a comprehensive statement about seroprevalence in dogs and horses is yet to be made, testing for multiple antibodies against *B. burgdorferi* s.l. should be considered.

Most animals enrolled in the present study had received antiparasitic treatments and, accordingly, in a recent study on animals involved in AAI, the owners of the dogs reported a regular administration of antiparasitic treatments. In the same study, although all horse owners reported that they occasionally administered parasiticides, no horse (nor dog) had ectoparasite infestation at the time of inclusion [20]. A recent survey on VBDs in dogs in Italy showed that almost half (i.e., 47.1%) of the dogs included were irregularly treated for ectoparasites [45], whereas the percentage of the dogs studied here that were treated irregularly or not at all with ectoparasiticides throughout the year was much lower. Thus, the data obtained here suggest that dogs included in AAI programs and/or living or in contact with vulnerable people receive a generally greater attention regarding ectoparasite treatments. On the other hand, the finding of ticks in dogs and horses subjected to antiparasitic treatments only in summer or irregularly, i.e., “when necessary” at the initiative of the owners, underlines that this approach is unsuccessful and that animals treated randomly are at risk of infection with ticks and related TBDs. In addition, the presence of ticks in two dogs in which the owners reported year-round treatment with ectoparasiticides indicates that in some cases, owners may have little knowledge of the characteristics of ectoparasiticides (i.e., speed of kill, duration of efficacy, efficacy against different ectoparasite species) and that efforts by veterinarians are needed to improve control measures [45].

The only statistically significant association shown here is not surprising, as a history of tick infestation is the basis for the seropositivity for at least one TBD. The absence of a statistical correlation between the presence of clinical signs and seropositivity rates found for *A. phagocytophilum* or *B. burgdorferi* s.l., along with the high frequency of non-specific clinical signs, confirm that equine and canine anaplasmosis and borreliosis may be challenging and elusive in clinical settings. Among sick animals, clinical signs related to the locomotor system were very frequent according to the literature [7,9,13,15]. Thus, when animals are presented with lameness, joint effusion/swelling, stiff gait or arthritis, *A. phagocytophilum* and *B. burgdorferi* s.l. should be included in the differential diagnosis. On the other hand, the fact that half of the seropositive animals was clinically healthy and had no history of clinical signs related to TBDs speaks to the need for continual epidemiological surveillance.

Although infected animals do not directly infect humans and the detection of antibodies in the serum of animals included in this study does not necessarily mean that these animals have an acute infection, it indicates that they were once infected and thus are or have been epidemiological sentinels and/or a potential source of infection for ticks. It should be also considered that animals and humans may develop antibodies also toward non-pathogenic or non-zoonotic strains of *A. phagocytophilum*.

Animals with active infections by *A. phagocytophilum* and *B. burgdorferi* s.l. favor the circulation of these bacteria among tick populations, increasing the possibility of TBDs transmission from an infected tick to humans. Thus, the routine testing of animals that are in close contact with humans should generally be a priority under an epidemiological standpoint, even more so when living with immunocompromised persons, children, or elderly people, in whom anaplasmosis and borreliosis can cause severe clinical pictures and may be fatal [54,55,56]. In clinical settings, seropositivity to *A. phagocytophilum* and/or *B. burgdorferi* should be interpreted with caution, and any treatment option should be evaluated case-by-case by the curing veterinarian. The present results suggest that the zoonotic potential of *A. phagocytophilum* and *B. burgdorferi* s.l. and the risk related to TBDs in general may also be underestimated by owners of animals in contact with people at risk of developing a severe disease after the bite of an infected tick. This is suggested by (i) the number of animals with a history of tick infestation, (ii) the presence of ticks found on the animals on clinical examination and by (iii) the number of animals that were not subjected to any kind of antiparasitic treatment.

Climatic and ecological changes favor the spread of vectors competent for the transmission of TBDs, including *Ixodes ricinus*, which can efficiently transmit both *A. phagocytophilum* and *B. burgdorferi* s.l. [57,58,59]. As the risk of zoonotic TBDs increases, veterinarians should be aware of current and changing epidemiological scenarios and include anaplasmosis and borreliosis in the list of differential diagnoses when animals are presented with non-specific clinical signs of apparently unknown origin. These and previous data [20,45] indicate that owners may not adequately perceive of the importance of antiparasitic treatments, resulting in inefficient protection of their animals and increased spread of zoonotic pathogens. Up-to-date epidemiological knowledge and adequate control plans and routine checks are critical for preventing zoonotic TBDs infections. From this perspective, veterinarians are encouraged to raise awareness among horse and dog owners to reduce the health risk to both animals and humans, especially when the latter belong to vulnerable groups.

## Figures and Tables

**Table 1:** Number of dogs (*n* = 75) with clinical signs and laboratory alterations compatible with canine borreliosis and/or anaplasmosis observed during the clinical examination or reported in the medical history.

Clinical Signs and Laboratory Alterations	Visit *n* (%)	History *n* (%)
Lameness	18 (24)	3 (4)
Lethargy	16 (21.3)	2 (2.7)
Diarrhea	15 (20)	6 (8)
Dys-/anorexia	10 (13.3)	2 (2.7)
Arthritis	9 (12)	1 (1.3)
Musculoskeletal pain	5 (6.7)	−
Coagulation disorders	4 (5.3)	−
Fever	4 (5.3)	−
Polydipsia	4 (5.3)	1 (1.3)
Vomit	4 (5.3)	4 (5.3)
Cough	3 (4)	−
Neurological signs	3 (4)	−
Weight loss	2 (2.7)	−
Anemia	2 (2.7)	−
Lymphopenia	2 (2.7)	−
Increased alkaline phosphatase (ALP)	2 (2.7)	−
Thrombocytopenia	2 (2.7)	1 (1.3)
Polypnea	1 (1.3)	−
Joint pain	1 (1.3)	−
Keratitis	1 (1.3)	−
Lymphopenia	1 (1.3)	−
Lymph adenomegaly	1 (1.3)	−
Neutrophilia	1 (1.3)	−
Increased gamma-glutamyl transferase (GGT)	1 (1.3)	−
Body cavity effusions	1 (1.3)	−
Hypergammaglobulinemia	1 (1.3)	−
Hyperbilirubinemia	1 (1.3)	−
Uveitis	1 (1.3)	−
Difficulty to walk	1 (1.3)	−
Hematochezia	−	1 (1.3)
Hypoalbuminemia	−	1 (1.3)

*n* = number of dogs presenting the clinical sign.

**Table 2:** Number of horses (*n* = 75) with clinical signs and laboratory alterations compatible with equine borreliosis and/or anaplasmosis observed during the clinical examination or reported in medical history.

Clinical Signs and Laboratory Alterations	Visit *n* (%)	History *n* (%)
Weight loss	20 (26.7)	1 (1.3)
Lethargy	13 (17.3)	−
Jaundice	12 (16)	−
Lameness	12 (6)	1 (1.3)
Weakness	11 (14.7)	1 (1.3)
Inappetence	11 (14.7)	−
Fever	11 (14.7)	1 (1.3)
Colic	10 (13.3)	2 (2.7)
Uveitis	10 (13.3)	−
Stiff gait	10 (13.3)	−
Diarrhea	8 (10.7)	−
Epiphora	6 (8)	1 (1.3)
Uveitis	4 (5.3)	−
Limb swelling	3 (4)	1 (1.3)
Neurological signs	2 (2.7)	−
Hind paresis	2 (2.7)	−
Blepharitis	2 (2.7)	1 (1.3)
Joint effusion	2 (2.7)	1 (1.3)
Pale mucous membranes	2 (2.7)	−
Hind limb edemas	2 (2.7)	−
Sheath edema	1 (1.3)	1 (1.3)
Anemia	1 (1.3)	−
Hyperbilirubinemia	1 (1.3)	−
Ventral edema	1 (1.3)	−
Poor performance	1 (1.3)	1 (1.3)
Epistaxis	1 (1.3)	−
Cough	1 (1.3)	−
Nasal discharge	1 (1.3)	−
Ataxia	1 (1.3)	−

*n* = number of horses presenting the clinical sign.

**Table 3:** Details of the ELISAs and immunoblots used in the study.

Sample Origin	Type of Immunoassay; Immunoglobulin Class (Ig)	Antibodies Tested	Antigen	Name of Test Kit from EUROIMMUN *
Dog	ELISA; IgM	*Borrelia burgdorferi*, *Borrelia afzelii*, *Borrelia garinii*	Antigen mixture containing cultured solubilized *Borrelia burgdorferi* sensu stricto, *Borrelia afzelii*, and *Borrelia garinii*	Anti-*Borrelia* ELISA Dog (IgM)
Dog	ELISA; IgG	*Borrelia burgdorferi*, *Borrelia afzelii*, *Borrelia garinii*	Antigen mixture containing cultured solubilized *Borrelia burgdorferi* sensu stricto, *Borrelia* afzelii, and *Borrelia garinii*	Anti-*Borrelia* ELISA Dog (IgG)
Dog	ELISA; IgG	*Borrelia* VIsE antigen	VIsE	Anti-VIsE ELISA Dog (IgG)
Dog	ELISA; IgG	*Anaplasma phagocytophilum*	Recombinant and purified antigen from *Anaplasma phagocytophilum*	Anti-*Anaplasma phagocytophilum* ELISA Dog (IgG)
Dog	Immunoblot; IgM	*Borrelia* antigens	P100; p41 and p39; OspC; p21; p18	Anti-*Borrelia* EUROLINE Dog (IgM)
Dog	Immunoblot; IgG	*Borrelia* antigens	VIsE, p100, p41 and p39, OspA, OspC, p21, p18	Anti-*Borrelia* EUROLINE Dog (IgG)
Dog	Immunoblot; IgG	*Borrelia burgdorferi* sensu lato, *Anaplasma*, TBE virus	VIsE; OspC (p25); MSP-2; gpe[TBEV]	EUROLINE Tick-Borne Profile 1 Dog (IgG)
Horse	ELISA; IgG	*Anaplasma phagocytophilum*	Recombinant and purified antigen from *Anaplasma phagocytophilum*	Anti-*Anaplasma phagocytophilum* ELISA Horse (IgG)
Horse	ELISA; IgG	*Borrelia burgdorferi* sensu lato	Antigen mixture containing cultured solubilized *Borrelia burgdorferi* sensu stricto and *Borrelia afzelii*	Anti-*Borrelia* ELISA Horse (IgG)
Horse	Immunoblot; IgG	*Borrelia burgdorferi* sensu lato, *Anaplasma*, TBE virus	VIsE; OspC p25; p100; MSP-2; gpE[TBEV]	EUROLINE Tick-Borne Profile 1 Horse (IgG)
Horse	Immunoblot; IgG	*Borrelia* antigens	DbpA; VlsE Ba and VlsE Bb; L-Bb; p100; p58; p39; OspA; OspC; p18	Anti-*Borrelia* EUROLINE Horse (IgG)

Abbreviations: DbpA: purified recombinant protein DbpA; gpE [TBEV]: part of the glycoprotein E of the TBEV strain Neudoerfl produced in bacteria; L-Bp: lipids from Bb extracted from the membrane fraction; MSP-2: recombinant MSP-2 from *A. phagocytophilum*, purified by affinity chromatography; OspA: purified recombinant protein OspA (p31); OspC: recombinant highly specific OspC advance from different *Borrelia* genospecies purified by affinity chromatography; p100: purified recombinant protein p100; P18: recombinant highly specific protein p18 purified by affinity chromatography; P21: recombinant highly specific protein p21 purified by affinity chromatography; p41 and p39: purified recombinant p41 (flagellin) and p39 (bmpA); p58: purified recombinant protein p58; VIsE: recombinantly produced and highly purified variable major protein-like sequence, expressed from *Borrelia*; VlsE Ba and VlsE Bb: purified recombinant VlsE antigen from *Borrelia afzelii* (Ba) and *Borrelia burgdorferi* (Bb). * The sensitivity and specificity of each assay are listed in the respective test instructions.

**Table 4:** Number of dogs and horses seropositive for *Anaplasma phagocytophilum* and *Borrelia burgdorferi* s.l.

Dogs		Horses	
Positivity	*n*. (%)	Positivity	*n*. (%)
Seropositive for at least one pathogen	13 (8.7)	Seropositive for at least one pathogen	19 (12.7)
*Anaplasma phagocytophilum*	1 (0.7)	*Anaplasma phagocytophilum*	12 (8)
*Borrelia burgdordferi* s.l.	12 (8)	*Borrelia burgdorferi* s.l.	10 (6.7)

**Table 5:** Dogs seropositive for antibodies against *Anaplasma phagocytophilum* and/or *Borrelia burgdorferi* s.l. detected in Italy and relative presence of signs detected during clinical examination and/or in medical history.

Clinical Picture	*Anaplasma phagocytophilum* (*n*.)	*Borrelia burgdorferi* s.l. (*n*.)
No clinical signs	1	7
Lameness + arthritis	−	1
Diarrhea	−	1
Lethargy + polydipsia *	−	1
Lethargy, diarrhea, anorexia, fever	−	1
Cough	−	1

*n*—number of animal seropositive for each pathogen; *—present both on examination and in medical history.

**Table 6:** Horses seropositive for antibodies against *Anaplasma phagocytophilum* and/or *Borrelia burgdorferi* s.l. detected in Italy and relative presence of signs detected during clinical examination and/or in medical history.

Clinical Pictures	*Borrelia burgdorferi* s.l. (*n*.)	*Anaplasma phagocytophilum* (*n*.)	*Borrelia burgdorferi* s.l. *+ Anaplasma phagocytophilum* (*n*.)
No clinical signs	5	2	1
Epiphora	−	−	1
Lameness	−	1	−
Fever	1	−	−
Stiff gait + lameness	−	−	1
Stiff gait + joint effusion	−	1	−
Colics + diarrhea	−	1	−
Limb swelling + weight loss * + fever * + colic *	−	1	−
Fever + jaundice	−	1	−
Epistaxis	1	−	−
Lethargy	−	1	−
Inappetence	−	1	−

*n*—number of animal seropositive for each pathogen; *—present in the medical history.

**Table 7:** Results of the statistical analysis—binomial logistic regression.

	*p*-Value	Odds Ratio	95% CI
Presence of clinical signs	0.960	0.964	(−1.465/1.392)
Antiparasitic treatments	0.943	0.904	(−2.878/2.676)
Tick infestation (clinical visit)	0.607	0.453	(−3.814/2.230)
Tick infestation (history)	0.027	7.398	(0.222/3.780)
Other ectoparasite infestation	0.993	1.90	(−3270.419/3239.467)
Cohabitation with other animals	0.759	0.725	(−2.373/1.731)
Pet therapy activity present	0.445	1.845	(−0.958/2.182)
Permanently outdoor (housing)	0.904	1.134	(−1.919/2.171)
Mixed (housing) *	0.259	0.321	(−3.110/0.836)

* Dogs: spending half of the time in yard and half of the time indoor; horses: referred to those stabled in boxes.

## Data Availability

All the data generated are described in the present article.
